# Supplementary figures and images for: Adult Botulism of Unknown Source with Post-Toxin Anti-GQ1b Antibodies: Implications for Molecular Mimicry—A Case Report
Source: Neurol Int. 2025 Dec 29;18(1):8. doi: 10.3390/neurolint18010008 (PMC12844876; doi:10.3390/neurolint18010008)

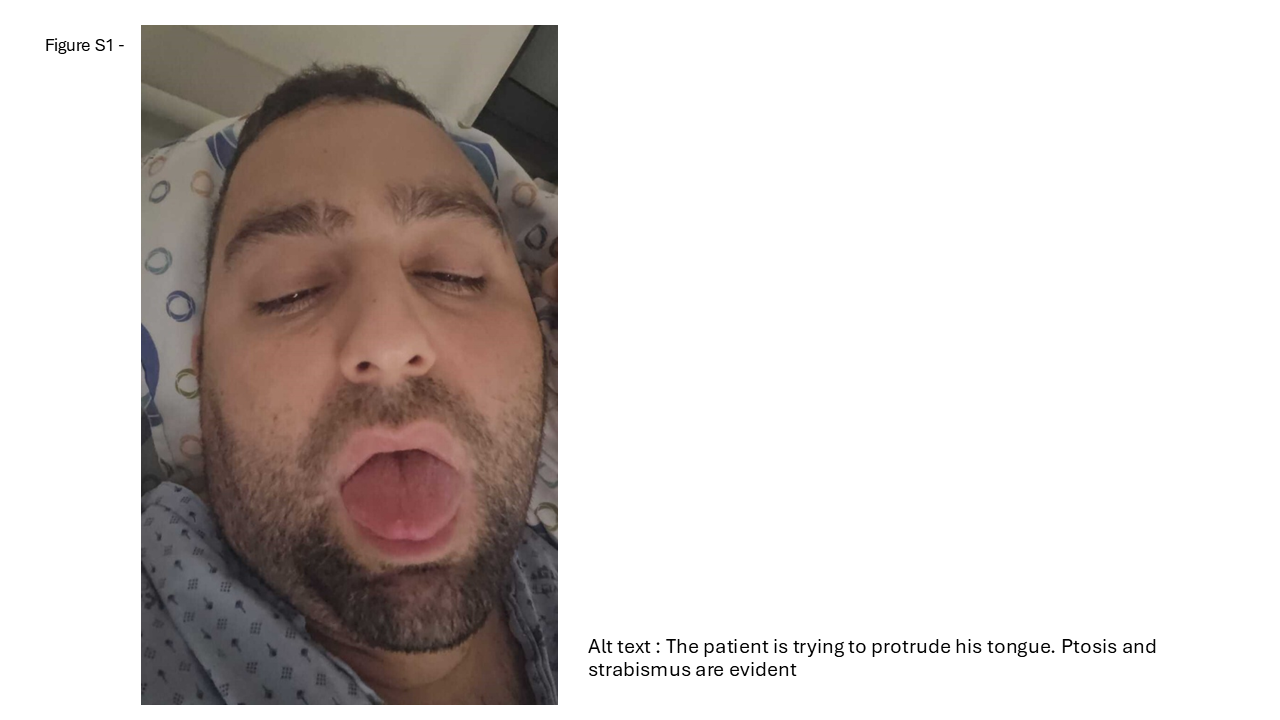

Supplement: Supplementary file 1 [file neurolint-18-00008-s001.zip › neurolint-3923994-figure S1.png]
